# Evaluation and spatial agglomeration analysis of the green competitiveness of China’s manufacturing industry at the provincial level

**DOI:** 10.1371/journal.pone.0246351

**Published:** 2021-03-30

**Authors:** Gang Li, Yi Yang, Xuming Lou, Yajie Wei, Sifeng Huang

**Affiliations:** School of Economics and Management, Xi’an University of Posts and Telecommunications, Xi’an, China; Institute for Advanced Sustainability Studies, GERMANY

## Abstract

The pressures to maintain a low-carbon economy makes green competitiveness (GC) a significant issue in China. It has been found that the development of Internet and E-commerce contributes significantly to GC of regional economy, yet there are still lack of quantitative investigation on their effects, which can be used to further guide the economic development. Therefore, this study constructs a new evaluation index for the green competitiveness of the regional manufacturing industry in China by introducing Internet application indicators and E-commerce development indexes into its evaluation system. The results show Jiangxi and Gansu moved upward significantly in the GC ranking over the period. The development of the Internet and E-commerce has the most significant impact on GC of regional manufacturing. The lack of green manufacturing capabilities and green innovation drivers led to a decline in the GC ranking of Liaoning, Tianjin, Guangxi and Heilongjiang. Finally, this study uses Moran’s *I* index to investigate the spatial agglomeration effect of the green development of the manufacturing industry at the province level. The results show an increase in the GC of China’s regional manufacturing industry, and the GC of manufacturing industry shows a significant agglomeration effect. Based on the above conclusions, the proposal to promote the balanced development of the GC of the manufacturing industry is proposed.

## 1. Introduction

Nowadays, green manufacturing has become a necessary development mode for the manufacturing industry (MI) in various countries [1]. Under the traditional development model in China, the MI emits substantial amounts of harmful gases and solid particulates in the production process, which resulted in environmental pollution, poor environmental optimization, high carbon emissions, and other issues [2]. China must adapt to changing economic development methods, promoting the low-carbon development mode, adopting low-carbon production [3], vigorously developing green competitiveness (GC), and enhancing the GC advantage of the MI, to prevent such environmental pollution. China’s MI in the province level is focusing on green environmental protection in the development process and vigorously advocating the low-carbon development model to enhance GC. To quantitatively analyze the GC, linear statistical method can be used for calculation of the fair value, combining with the payment level of environmental governance and the quality benefit level [4]. Furthermore, with the rapid development of the Internet economy in China, the production and sales modes of the MI are changing [5], especially in the promotion of energy-saving and environmental protection. Thus, the GC of the provincial regions is undergoing tremendous change, creating an urgent need in academic circles for new definitions and fresh evaluations.

In recent years, it is noticeable that Internet and E-commerce industries have been playing an increasingly important role in economy and significantly influencing regional GC, yet there is still lack of quantitative investigation about these effects. A clear cognition on their effects and relationship to the MI development will be very helpful in evaluation of comprehensive regional development. Aiming at this problem, this study contributes to the evaluation of green competitiveness of the regional manufacturing industry (GCRMI) by focusing on the important new indicators that have recently emerged from the provincial industry-level evidence (in official statistics, data are processed at the provincial level).

Moreover, the GC of China’s MI is also a problem of regional balance. Interregional equilibrium is an effective way to promote the green development of the regional MI in China, especially in the less developed provinces [6]. It is also an essential requirement of the strict restraint mechanism for harmonious development in the regional economy and environment. It can attract economic activities to a particular region [7,8]. There have been a few studies on the relationship between manufacturing agglomeration and environmental pollution, indicating that it is of the inverted “N” type, and the degree of manufacturing agglomeration in China is at a low-to-medium level, which causes high environmental pollution [9]. However, the investigations in this aspect are still far from sufficient. This study also contributes to the analysis of the spatial agglomeration effect of the GCMI in the provincial regions. We investigate whether a spatial correlation exists for the GCRMI and the characteristics of any spatial agglomeration effect. As GC is also a significant international concern, we believe the results and conclusions in this study will provide beneficial reference for research and development in other countries all around the world.

## 2 Literature review

### 2.1 Green manufacturing and GC

Green manufacturing is regarded as a philosophy rather than a standard or process. Its main goal is sustainability, and MI uses it to minimize waste and pollution through product and process design [10]. Research on the Indian MI has provided a road map and performance measures for green manufacturing practice [11].

The GC issue is now a focus in both research field and industries all around the world. The United States of America has put forward the Green New Deal so as to promote a better economic growth in a world-wide range [12]. The European Union (EU) is considering to introduce a Border Carbon Adjustment (BCA) to ensure that the price of imports into the EU more accurately reflects the environmental costs of their carbon content, and the core concern of this plan is on green manufacturing and industrial competitiveness [13]. Regional investigation on GC is widely used to reflect the imbalanced development in countries. For example, in investigation of GC in the Polish regions, Kasztelan divided it into for groups, each of which has it unique characteristics [14]. In India, GC is considered as not only an economic index but also an indicator of culture development [15]. Great importance has been laid to green manufacturing in India, and it is found that small and medium sized enterprises can play a critical role in GC [16].

### 2.2 Drivers and factors of GC

Many scholars believe that the government’s green regulations have played a positive role in promoting the GC of enterprises, something that was bound to be valued and implemented by many government decision-makers [17]. Many studies have examined the incentive mechanism of environmental regulation on technological innovation and green productivity improvement, finding that environmental regulation has a positive and indirect impact on the promotion of green productivity [18]. Research shows that the relationship between sustainable manufacturing practices and the competitive capabilities of Egyptian SMEs is positive and significant [19]. Scholars have shown that green development was unavoidable in the MI, presenting corresponding implementation paths and strategies [17].

Even though the environmental regulation, market competition and benefits have been drivers of GC [16], many scholars have focused on the performance and profits of green manufacturing and GC alone, rather than on their role as drivers [20–22]. Economic pressures and crises have forced companies to adopt green manufacturing for financial benefits; they have optimized resources and energy to decrease costs, inadvertently formulating GC [23]. Carbon barriers, such as the imposition of carbon tariffs and the promotion of carbon labels, are international drivers that forced companies to decrease energy consumption and carbon emissions.

We can conclude from previous research that several factors influence GC. Sustainable development strategies based on environmental concerns have had an essential impact on the GC of companies [23]. Taking a comprehensive view of strategic green marketing and its impact on environmentally driven competitive advantage, Papadas et al. investigated the moderating role of internal green marketing actions in the development of sustained competitive advantage. They revealed a significant interplay between strategy and people, which helps create an environmentally driven competitive advantage [24]. In response to the requirements of GC development, green innovations have emerged in the current research realm, forcing companies to adopt new sustainable tactics [23]. Green innovation is effective in overcoming pressure from customers, competitors, and regulators. The three main categories of green innovation (green product innovation, green process innovation, and green managerial innovation) have been positively associated with environmental performance and competitive advantage [20,22,25]. The effect of green technology innovation was examined based on an empirical study of 209 listed companies in the heavily polluting MI. The findings indicated that green process innovation had a positive impact on green product innovation. Both of them could improve a firm’s financial performance. Green image (as green competitiveness) moderated the relationship between green product innovation and economic performance [26].

The Internet economy is another factor in GC. Information and communication technologies have led to the emergence of the “collaborative production” mode, through which Internet companies not only buy materials but also share storage resources, computing resources, machines, designs, and other manufacturing resources. Transformed from the business-to-business (B2B) mode of electronic commerce, the “industrial cloud platform” incorporates regional factories and technological resources, allowing companies to obtain various services and support at low rental costs [5]. Thus, the B2B level and Industrial Internet have driven GC. Recent research has proved the advantages of lower cost, higher convenience, and increased applicability of critical manufacturing processes of the industrial Internet-of-Things, that will lead to the GC goals of energy-saving, low emissions, and customer cost savings.

Furthermore, the Internet-of-Things is an emerging technology for smart cities, which connects various digital devices through the Internet, providing multiple innovative facilities in areas from academia to industry [2]. Fifth-generation wireless transmission technology is likely to give a significant impetus to the industrial Internet-of-Things, helping companies achieve more up-to-date GC [27]. Table 1 shows the drivers and factors of GC. Along with the economy development, the Internet application and E-commerce development have become increasingly important drivers in GC. And it is very reasonable and meaningful to embed these two factors in the evaluation of GC so as to adjust to the rapid development of economy and the society.

**Table 1:** Drivers and factors of GC.

Drivers and Factors of GC	References
The government’s green regulations	[17,28]
Environmental regulation	[17,18]
Low-carbon economy	[19,29]
The market competition and benefits	[16]
The imposition of carbon tariffs	[23]
The promotion of carbon labels	[23]
Internal green marketing actions	[24]
The green innovations	[22,23,25]
The Internet economy	[2,5,27]

### 2.3 Evaluation of GC

Evaluation of GC is the essential issue in all the relative researches. Many studies on strategy and competition have made long-term and in-depth examinations of the competitiveness of enterprises or regions. Factor analysis has been used to evaluate competitiveness in the MI [30] by assessing the competitiveness of cross-border E-commerce enterprises within the MI. Cross-border marketing ability and technology adoption ability have been proposed as important factors affecting the competitiveness of cross-border E-commerce enterprises in the MI [31].

Simultaneously, GC, as a new division of competitiveness, is gaining popularity among scholars. Studies have developed a toolbox (the Greenometer) to assess the level of the greenness of manufacturing companies [32]. Green competitiveness evaluation index for in iron and steel enterprises was established, combining analytic hierarchy process and entropy value method, green competitiveness evaluation model of enterprises can be realized [31].

As research in this area develops, GC has extended from the enterprise to manufacturing. Scholars have focused on evaluating the GCMI, mostly using provincial panel data and a variety of calculation methods. Green development along the Belt and Road is related both to the success of the construction along the Belt and Road, and further, the sustainable development of the countries [33]. Zhai and Lu constructed an index system comprising the “green production factor” and “product competitiveness” to study the competitiveness of industrial enterprises in Liaoning Province. They used factor analysis to evaluate the performance of industrial transformation and upgrading in the province and found that the low competitiveness of products became the biggest obstacle to the transformation and upgrading of manufacturing [34]. Using factor analysis, Wu and Jing [35] studied the GC of the county economy under the concept of green development in Hubei Province, formulating policies such as differentiated county green development policies, and cultivating a county green social system. A regional GC index was developed that applied correlation analysis, fuzzy rough sets, and entropy weight methods to select and analyze 21 indicators. These indicators included measures of competitiveness of natural resources, ecological environment, energy consumption and energy-saving, economic and social sustainability, and human health. Based on the resulting index, the GC spectrum was divided into three levels (light green, medium green, and dark green) to explore changes in 30 provinces of China during the period 2004 to 2014. Spatial differences in GC vary from high to low in a westerly direction [29].

The green development of regional MI in China may lead to the use of green information technology and the promotion of clean energy reform in the MI through further development of the Internet. This prospect has provided a new direction, expanded horizons, and marked out new paths for green reform [36]. Consequently, Internet infrastructure and applications have been incorporated into the GC evaluation system for regional MI. The system now comprises several indicators, including manufacturing market share, manufacturing market optimization index, and Internet penetration [37]. See Tables 2 and 3.

**Table 2:** Evaluation indexes of GC.

Evaluation index of GC	Reference
Carbon dioxide emissions per capita	[38]
Energy consumption rate per unit GDP	[35,38]
Investment index of urban environmental protection	[38,39]
Forest coverage	[35,38,39]
Proportion of added value of high tech industry in GDP	[35]
Comprehensive utilization rate of industrial solid waste	[39]
Per capita added value of manufacturing	[37]
Market share of manufacturing	[37,38,40]
Manufacturing market optimization index	[37]
Proportion of R&D expenditure in the main business income of regional manufacturing industry	[37]
Proportion of environmental protection expenditure in financial expenditure	[37,40].
Internet penetration rate	[37]
Number of patents for effective inventions in high-tech industries per 10,000 people	[37]
Proportion of cross border E-commerce sales revenue	[31]

**Table 3:** Research methods of GC evaluation.

Research method of GC evaluation	References
Quantitative evaluation	[38]
Factor analysis	[31,34,35,39]
Projection pursuit model based on genetic algorithm	[37]
Fuzzy rough set	[29,39]
Analytic hierarchy process	[39]
Entropy weight method	[29,39]
Correlation analysis	[29]

We can conclude from the above review that little work has been done on analyzing the GCMI, especially from the regional perspective. Similarly, no studies have examined the agglomeration analysis of GC at the provincial region level. Most studies have not examined the role of the Internet economy in green manufacturing, nor have they considered the levels of Internet applications as essential components in the evaluation index system of GC. Therefore, this study constructs a new evaluation index for the green competitiveness of the regional manufacturing industry in China by introducing Internet application indicators and E-commerce development indexes into its evaluation system.

## 3. Methodology

### 3.1. Process and methodology

**Fig 1:**
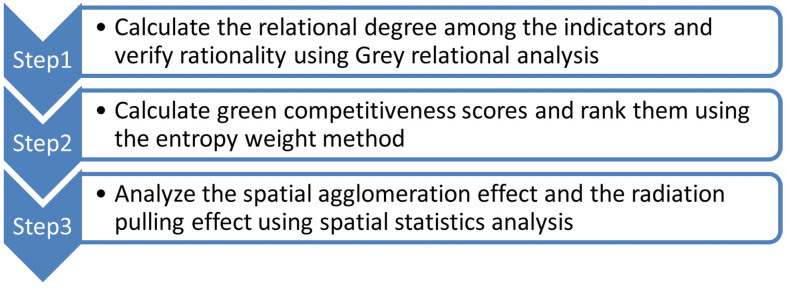
Process and methodology of this empirical research.

This study constructs an index system for the GCRMI and performs empirical research in three steps (Fig 1).

In Step 1, the Grey relational analysis method is used to calculate the relational degree among the indicators, which is then used to verify the rationality of the index system constructed in this study. In Step 2, the entropy weight method is used to calculate the indicators to obtain a GC score, and the 31 provinces in the country are ranked comparatively. In Step 3, spatial statistics analysis is used to investigate the spatial agglomeration effect of the development status of the GCRMI, and to analyze the radiation pulling effect among provinces.

### 3.2. Developing an evaluation index system

#### 3.2.1. The level of internet application

“Internet + manufacturing” is the core feature of the new round of technological revolution and industrial reform, and crucial for China to transform and upgrade its MI [41]. Not only is it convenient, efficient, and transformational, but it must also be clean and green [42]. The deep integration of cloud computing, Internet-of-Things, intelligent industrial robots, 3D printing, and so on, in the MI, can implement cleaner production and reduce the use of toxic and hazardous substances, thereby, enhancing green GC and sustainable development capacity in the MI [43].

The core of Internet-manufacturing operations still concerns enhancing the economic value of products. The Industrial Internet represents the most significant advances in the development of intelligent manufacturing [44]. Industrial Internet in the MI is a method to contain environment, economy, and social needs through five key factors: (1) sustainability credibility, (2) concern for environmental impact, (3) careful consideration of stakeholders, (4) resource efficiency, and (5) a holistic philosophy [45]. The Industrial Internet systematically embeds the Internet’s intelligent concept into the manufacturing green development system, which can improve both manufacturing efficiency and its GC in various markets [46]. So, the level of Internet application is one of the critical indicators of green competitiveness of the MI.

#### 3.2.2. E-commerce in the manufacturing industry

E-commerce based on Internet technology can drive online and offline interactions in the MI, and achieve the “four streams” of business flow, logistics, capital flow, and information flow. It significantly enhances the probability of enterprises entering the export market and promotes the expansion of export volume. It enables more substantial international competitiveness for the MI [47].

In E-commerce, transaction activities mainly occur in the B2B environment, B2B companies face strong government regulations and public pressure due to their products’ significant impact on the environment and society. The B2B manufacturing industry’s characteristics influence how users and customers may be leveraged [45]. According to the industry-level input-output matrix empirical results of the BRIC countries from 1995 to 2009, companies have significantly improved environmental performance by using cleaner inputs and importing greener and more environmentally friendly inputs from developed countries. When the domestic industry does not have sufficient support for green services, it is feasible to achieve environmental benefits through trade liberalization to purchase clean procurement of raw material [48]. The B2B field has also begun to improve sales by applying manual labor or big data [49]. It is foreseeable that its greenness will be further enhanced [50].

The MI is mostly at the center of B2B and has the unique advantage of integrating the supply chain. Therefore, the B2B transaction volume of the MI represents the green degree of the entire industry to some extent.

#### 3.2.3. New evaluation index system for GC

This study has been greatly facilitated by the evaluation system for the GCRMI constructed by Lin Li and Zu Wang in their paper, “Evaluation and dynamic comparison of green competitiveness of the regional manufacturing industry” [37]. Building on their contribution, this study constructs a new evaluation index system. The existing evaluation index system contains too many similar indicators, some of which do not reflect the characteristics of new production factors (such as “Internet +” activities) and do not accurately characterize the development status of the GCRMI. Therefore, this study introduces an evaluation index based on E-commerce development, average B2B transaction volumes, and Internet penetration rates to analyze the green market competitiveness of the MI and the GC of production factors under the new development mode, “Internet + manufacturing.”

To avoid measuring errors caused by using the same indicators for a whole region, this study introduces the indicators of Per capita expenditure on science and technology in MI and the Number of patents for effective inventions in high-tech industries per 10,000 people. These indicators allow us to study the green innovation ability of regional MI. In addition, the introduction of Per capita sales revenue of high-tech new products and Per capita added value of manufacturing industry in high tech industry reflects the high-tech value output of the MI; thus, representing GC. With the vigorous promotion of production and environment development, and considering the high levels of energy consumption and pollution, the GCRMI also needs to embody the characteristics of environmental friendliness. Therefore, this study introduces indicators such as waste treatment utilization rates and energy consumption reduction rates for the regional gross product and constructs a new evaluation index system for the GCRMI.

Table 4 shows the evaluation index system for GC. It includes three first-level indexes: green market competitiveness, green manufacturing capability, and green innovation capability. Each first-level index contains several second-level indexes.

**Table 4:** Evaluation index system of the GC of the manufacturing industry.

**First-level index**	**Second-level index**
**Green market competitiveness (A)**	Per capita added value of regional manufacturing industry (Aa)
	Proportion of regional manufacturing industry assets in national assets (Ab)
	Regional manufacturing industry market share (Ac)
	Regional manufacturing industry market optimization index (Ad)
	Regional manufacturing industry fixed assets novelty coefficient (Ae)
	Per capita sales revenue of high-tech new products (10,000 yuan) (Af)
	Per capita added value of manufacturing industry in high tech industry (Ag)
	E-commerce transaction volume per capita (Ah)
	Average transaction volume of B2B (Ai)
	Internet penetration rate (Aj)
	E-commerce development index (Ak)
**Green manufacturing capacity (B)**	Sulfur dioxide emissions (10,000 tons) from manufacturing industry per value added of 100 million yuan (Ba)
	Sewage discharge (10,000 tons) of manufacturing industry per value added of 100 million yuan (Bb)
	Utilization rate of industrial waste treatment (Bc)
	Proportion of environmental protection expenditure to fiscal expenditure (Bd)
	Energy consumption reduction rate of 10,000 yuan regional gross product (Be)
**Green innovation capability (C)**	Proportion of R&D personnel in regional manufacturing industry (Ca)
	Proportion of R&D expenditure in the main business income of regional manufacturing industry (Cb)
	The proportion of high-tech manufacturing employment in manufacturing employment (Cc)
	Per capita expenditure on science and technology (10,000 yuan) (Cd)
	Number of patents for effective inventions in high-tech industries per 10,000 people (Ce)
	Proportion of high-tech investment in social investment (Cf)

### 3.3. Entropy weight Grey relational analysis model

The entropy weight Grey relational analysis method is a combination of the entropy value method and the Grey relational analysis method. Given the shortcomings of these two methods, the expected results cannot be achieved using either method alone. The Grey relational analysis method was originally used to study the relationship between the particle content of phosphorus slag and cement strength. When small samples provide inaccurate and incomplete information, a Grey correlation analysis can be used to obtain more accurate results by calculating the degree of correlation between the indicators. However, the evaluation accuracy of the Grey relational analysis model is too low for satisfactory measurement. Therefore, to improve accuracy, this study combines it with the entropy value method, which can reduce the role of subjective factors in the process of empowerment, and it is more scientific, accurate, and robust.

#### 3.3.1. Entropy weight model

The weight of the GC index is obtained using the entropy weight method, and the weight of the index reflects most of the original information it contains [51]. The specific steps are as follows:

1. Obtaining the decision matrix. The number of provinces and municipalities participating in the evaluation is a, and the number of evaluation indicators is b. A specific index is expressed by k_ij_, the value of the jth evaluation index of the ith evaluation of province and municipality. Thus, a decision index matrix of a × b can be obtained as follows:
  $$K=\left(k_{\mathrm{ij}}\right)\;a\times b=\left[\begin{array}{cccc} k_{11} & k_{12} & & k_{1b}\\ k_{21} & k_{22} & \cdots & k_{2b}\\ & \vdots & \ddots & \vdots \\ k_{a1} & k_{a2} & \cdots & k_{\mathrm{ab}} \end{array}\right],\;i=1,2,3\ldots ,a;j=1,2,3\ldots ,b$$ (1)
2. Standardization of the decision matrix. Because the matrix aggregate index is not standard, and because of the existence of dimensional factors, it is necessary to standardize the decision-making matrix.
  $$q_{\mathrm{ij}}=\frac{k_{\mathrm{ij}}}{\sum_{i=1}^{a}k_{\mathrm{ij}}}(j=1,2,3\ldots ,b)$$ (2)
  In Formula (1), the value range of q_ij_ is [0,1], and q_ij_ denotes the standardized value of index j of the ith province and municipality, forming a new decision matrix:
  $$Q=\left(q_{\mathrm{ii}}\right)\;a\times b=\left[\begin{array}{cccc} q_{11} & q_{12} & & q_{1b}\\ q_{21} & q_{22} & \cdots & q_{2b}\\ & \vdots & \ddots & \vdots \\ q_{a1} & q_{a2} & \cdots & q_{\mathrm{ab}} \end{array}\right],\;i=1,2,3\ldots ,a;j=1,2,3\ldots ,b$$ (3)
3. Computing the entropy value e_j_ of the jth index as follows:
  $$e_{i}=‐\frac{1}{\ln a}\sum_{i=1}^{a}\;q_{\mathrm{ij}}\;\ln q_{\mathrm{ij}}$$ (4)
  When q_ij_ is 0 or 1, q_ij_lnq_ij_ is 0.
4. Computing the coefficient of difference d_j_ of the jth index as follows:
  $$d_{j}=1‐e_{j}$$ (5)
  The index weight increases with the increase of d_j_ (i.e., the greater the value of d_j_, the greater the index weight).
5. Determine the weight of each index as follows:
  $$w_{j}=\frac{d_{j}}{\sum_{j=1}^{b}d_{j}}$$ (6)

#### 3.3.2. Grey relational degree model

1. By normalizing each index and normalizing matrix K = (k_ij_)_a×b_, we obtain the following:
  $$p_{ij}=\frac{k_{ij}-\underset{1\leq m\leq a}{\min}\left(k_{mj}\right)}{\underset{1\leq m\leq a}{\max}\left(k_{mj}\right)-\underset{1\leq m\leq a}{\min}\left(k_{mj}\right)}i=1,2,3\ldots ,a;j=1,2,3\ldots ,b$$ (7)
  In Formula (5), k_mj_ denotes the jth exponential value of the mth sample.
  This gives a new dimensionless matrix as follows:
  $$P=\left(p_{ij}\right)\;a\times b=\left[\begin{array}{cccc} p_{11} & p_{12} & & p_{1b}\\ p_{21} & p_{22} & \cdots & p_{2b}\\ & \vdots & \ddots & \vdots \\ p_{a1} & p_{a2} & \cdots & p_{ab} \end{array}\right]\;i=1,2,3\ldots ,a;j=1,2,3\ldots ,b$$ (8)
2. Formula (5) is normalized and a new sequence is obtained. It is a classification standard series recorded as K_0_. Calculating the Grey correlation coefficient [52], the correlation coefficient of sequence p_ij_(i) = (p_i1_, p_i2_…, p_ib_) on the jth index is
  $$\varsigma_{i}(j)=\frac{\left(\underset{1\leq i\leq a}{\min}\;\underset{1\leq j\leq b}{\min}\;|k_{0j}-p_{ij}|+\rho \underset{1\leq i\leq a}{\max}\;\underset{1\leq j\leq b}{\max}\left|k_{0j}-p_{ij}\right|\right)}{\left(|k_{0j}-p_{ij}|+\rho \underset{1\leq i\leq a}{\max}\underset{1\leq j\leq b}{\max}\left|k_{0j}-p_{ij}\right|\right)}i=1,2,3\ldots ,a;j=1,2,3\ldots ,b$$ (9)
  In Formula (6), let ρ =  0.5, the resolution coefficient. |k_0j_−p_ij_| is the absolute difference between index k_0_ and p(j); $\underset{1\leq j\leq b}{\min}\left|k_{0j}-p_{ij}\right|$ is the level 1 minimum difference; $\underset{1\leq i\leq a}{\min}\;\underset{1\leq j\leq b}{\min}\left|k_{0j}-p_{ij}\right|$ is the level 2 minimum difference; $\underset{1\leq j\leq b}{\max}\left|k_{0j}-p_{ij}\right|$ is the level 1 maximum difference; and $\underset{1\leq i\leq a}{\max}\;\underset{1\leq j\leq b}{\max}\left|k_{0j}-p_{ij}\right|$ is the level 2 maximum difference.
3. After optimizing the entropy weight, the comprehensive Grey correlation degree is as follows:
  $$G_{i}=\sum_{j=1}^{b}w_{j}\varsigma_{i}(j)i=1,2,3\ldots ,a;j=1,2,3\ldots ,b$$ (10)
  Finally, the correlation degree of the GC indicators can be evaluated objectively, according to the G_i_ value. The greater the G_i_ value, the higher the degree of correlation between GC indicators.
4. Calculation of GC of the provincial regional manufacturing industry. The GC of the manufacturing enterprises in all provinces and municipalities is calculated, and each province and municipality is ranked as follows:
  $$C_{i}=\sum_{j=1}^{b}w_{j}q_{\mathrm{ij}}$$ (11)

### 3.4. Data sources

This study selected 31 provinces and municipalities across the country, as research objects, and evaluated their GC by searching the corresponding index data. The industrial data in this study is mainly from the *Statistics Yearbook on Science and Technology Activities of Industrial Enterprises*, *China Statistics Yearbook on High Technology Industry*, *China Industry Statistics Yearbook*, *China Statistics Yearbook on Environment*, *China Energy Statistics Yearbook*, and *China Statistics Yearbook* (all in Chinese) from 2013 to 2018 [53–58].The Internet data is mainly from the *China Statistical Report on Internet Development* (in Chinese) from 2013 to 2018 [59]. *China E-commerce Development* I*ndex Report* (in Chinese) from 2013 to 2018 [60], *China E-commerce Market Data Monitoring Report* (in Chinese) from 2013 to 2018 [61].

## 4. Results and discussion

### 4.1. Relevance analysis

The following three indicators represent the development degree of manufacturing efficiency of a province or municipality: Aa, Af, and Ag. The bigger the index value, the better the enterprise’s efficiency and development. However, no single factor determines the development of regional manufacturing, and the corresponding evaluation indicators are diversified. Therefore, we can calculate the correlation degree of each index, especially the correlation degrees with the revenue of MI, to judge the rationality of the GC index of the regional MI. The Grey correlation analysis is carried out between each index and the Aa, Ag, and Af. The results prove that the GC index system constructed in this study is reasonable (Tables 5–7).

**Table 5:** Index correlation degrees with Aa.

Index	Correlation degree						Average	Rank
	2013	2014	2015	2016	2017	2018		
Ab	0.894	0.887	0.900	0.894	0.895	0.844	0.8857	13
Ac	0.880	0.873	0.886	0.879	0.875	0.867	0.8767	16
Ad	0.934	0.929	0.934	0.925	0.925	0.832	0.9132	3
Ae	0.905	0.896	0.903	0.897	0.898	0.895	0.8990	9
Af	0.805	0.846	0.864	0.871	0.877	0.873	0.8560	21
Ag	0.871	0.868	0.889	0.890	0.890	0.879	0.8812	14
Ah	0.872	0.856	0.876	0.875	0.868	0.868	0.8692	17
Ai	0.879	0.856	0.864	0.859	0.864	0.864	0.8643	19
Aj	0.897	0.897	0.907	0.904	0.902	0.891	0.8997	7
Ak	0.924	0.879	0.877	0.932	0.916	0.929	0.9095	5
Ba	0.897	0.887	0.891	0.870	0.864	0.856	0.8775	15
Bb	0.892	0.888	0.901	0.902	0.899	0.887	0.8948	10
Bc	0.900	0.895	0.907	0.895	0.885	0.868	0.8917	12
Bd	0.908	0.893	0.902	0.903	0.900	0.891	0.8995	8
Be	0.913	0.906	0.897	0.900	0.884	0.856	0.8927	11
Ca	0.869	0.864	0.873	0.872	0.866	0.855	0.8665	18
Cb	0.920	0.918	0.925	0.924	0.925	0.916	0.9213	1
Cc	0.904	0.897	0.909	0.908	0.915	0.901	0.9057	6
Cd	0.909	0.904	0.913	0.915	0.919	0.914	0.9123	4
Ce	0.863	0.851	0.871	0.870	0.866	0.858	0.8632	20
Cf	0.915	0.910	0.923	0.919	0.912	0.911	0.9150	2

**Table 6:** Index correlation degrees with Ag.

Index	Correlation degree						Average	Rank
	2013	2014	2015	2016	2017	2018		
Aa	0.844	0.843	0.873	0.860	0.867	0.825	0.8520	10
Ab	0.863	0.861	0.878	0.861	0.872	0.808	0.8572	7
Ac	0.870	0.866	0.884	0.865	0.870	0.773	0.8547	9
Ad	0.832	0.829	0.853	0.829	0.835	0.798	0.8293	14
Ae	0.797	0.793	0.821	0.795	0.808	0.758	0.7953	19
Af	0.807	0.899	0.921	0.908	0.913	0.901	0.8915	2
Ah	0.887	0.866	0.872	0.855	0.870	0.845	0.8658	4
Ai	0.890	0.858	0.872	0.842	0.852	0.824	0.8563	8
Aj	0.816	0.840	0.859	0.828	0.843	0.788	0.8290	15
Ak	0.847	0.833	0.846	0.839	0.861	0.807	0.8388	12
Ba	0.784	0.779	0.808	0.778	0.784	0.715	0.7747	21
Bb	0.844	0.841	0.863	0.848	0.853	0.761	0.8350	13
Bc	0.791	0.795	0.832	0.804	0.822	0.742	0.7977	18
Bd	0.801	0.798	0.825	0.813	0.830	0.762	0.8048	17
Be	0.800	0.805	0.825	0.809	0.805	0.715	0.7932	20
Ca	0.866	0.862	0.877	0.856	0.861	0.825	0.8578	6
Cb	0.827	0.826	0.855	0.828	0.838	0.770	0.8240	16
Cc	0.849	0.846	0.867	0.844	0.853	0.803	0.8437	11
Cd	0.897	0.896	0.913	0.895	0.901	0.878	0.8967	1
Ce	0.885	0.874	0.888	0.868	0.870	0.838	0.8705	3
Cf	0.862	0.859	0.880	0.860	0.865	0.842	0.8613	5

**Table 7:** Index correlation degrees with Af.

Index	Correlation degree						Average	Rank
	2013	2014	2015	2016	2017	2018		
Aa	0.827	0.814	0.830	0.830	0.836	0.831	0.8280	10
Ab	0.822	0.845	0.854	0.843	0.843	0.830	0.8395	7
Ac	0.822	0.847	0.855	0.845	0.840	0.776	0.8308	9
Ad	0.839	0.795	0.805	0.789	0.786	0.808	0.8037	16
Ae	0.837	0.763	0.775	0.760	0.759	0.752	0.7743	19
Ag	0.853	0.897	0.912	0.903	0.903	0.908	0.8960	1
Ah	0.862	0.853	0.840	0.844	0.839	0.836	0.8457	6
Ai	0.860	0.845	0.840	0.825	0.825	0.822	0.8362	8
Aj	0.833	0.817	0.823	0.799	0.802	0.789	0.8105	14
Ak	0.852	0.811	0.815	0.815	0.827	0.816	0.8227	11
Ba	0.813	0.752	0.764	0.75	0.742	0.732	0.7588	21
Bb	0.826	0.824	0.829	0.822	0.817	0.767	0.8142	12
Bc	0.807	0.786	0.818	0.803	0.804	0.755	0.7955	17
Bd	0.833	0.756	0.775	0.772	0.777	0.771	0.7807	18
Be	0.838	0.778	0.781	0.771	0.754	0.720	0.7737	20
Ca	0.827	0.872	0.880	0.869	0.867	0.871	0.8643	4
Cb	0.844	0.796	0.818	0.805	0.805	0.776	0.8073	15
Cc	0.851	0.806	0.821	0.801	0.804	0.798	0.8135	13
Cd	0.842	0.856	0.879	0.880	0.873	0.889	0.8698	2
Ce	0.843	0.876	0.881	0.869	0.864	0.857	0.8650	3
Cf	0.829	0.845	0.858	0.854	0.855	0.862	0.8505	5

From Tables 5–7, we can see that in the period 2013 to 2018, the degree of correlation of each index obtained by Grey correlation analysis was greater than 0.75. The Grey correlation degree between most of the indicators was close to 1, which suggests that these indicators played a very large role in promoting the green development of manufacturing. Thus, the GC index system constructed in this study shows certain rationality.

From Table 5, we can see that the highest degree of correlation in the years 2013 to 2018 was between Aa and Cb. The degree of correlation with Cf was in second place. The degree of correlation with Ad was in third place. Cd was closely behind. The correlation degree with Af was the smallest. These results told that the introduction of high-tech talents into MI and the high investment in R&D affected the green development of regional MI. The intelligent optimization of the manufacturing market and the development of the Internet had driven the green development of MI.

Table 6 shows that Cd ranked the first. The degree of correlation with Af and with Ce was in the second and the third place. The results suggested that the development of high-tech industries should focus on investment high-tech products.

Table 7 shows that the degree of correlation with Ag was the highest, the degree of correlation with Cd came second. Ce came third. These results indicated that reasonable optimization of the manufacturing market and the scientific research funding were driving green development in manufacturing.

Tables 5–7 show a downward trend in the correlation between each index and the reference index. We can infer that with the diversification of GC evaluation indicators, the factors influencing the green development of regional manufacturing also tend to be diversified. The importance of a single index shows a downward trend, and the correlation coefficient decreases accordingly. The degree of correlation for Ad, Aj, Ak, Cd, Ce, and Cf were almost at the top of the rankings over the period. These results show that although the correlation between the indicators decreased overall, the indicators that had the most significant impact on the green development of manufacturing were still relatively concentrated. From the importance of these six indicators, it can be concluded that the introduction of high-tech industries and the rational utilization of waste were crucial to the development of regional manufacturing in the “high-tech” and “green” directions. Simultaneously, the degree of correlation between the Ak and Aa was in fifth place. The development of E-commerce has enlarged the green market of regional manufacturing, and the popularity of the Internet has become more closely related to the benefits of enterprises. The enhancement of the green innovation capability of regional MI has benefited from the development of “Internet +.” In sum, the index system is rational and practical.

### 4.2. GC score

This study used Excel spreadsheet software to calculate and process the data according to the weight of each index. The overall scores of each province were obtained and ranked (Table 8).

**Table 8:** GC score and ranking of MI in the 31 provinces, 2013–2018.

Year	2013		2014		2015		2016		2017		2018	
Province	Score	Rank	Score	Rank	Score	Rank	Score	Rank	Score	Rank	Score	Rank
Anhui	5.730	13	5.099	18	5.520	14	5.733	12	5.763	12	7.446	7
Beijing	8.573	6	8.855	5	8.786	5	8.537	6	8.827	5	8.772	3
Chongqing	5.559	15	5.516	13	5.857	11	5.871	11	6.100	11	6.995	9
Fujian	6.573	8	6.460	9	6.259	10	6.219	9	6.214	9	5.921	11
Gansu	3.298	30	3.251	29	3.268	27	2.980	28	3.294	25	3.751	23
Guangdong	12.323	1	13.250	1	13.108	1	13.227	1	13.450	1	12.970	1
Guangxi	4.678	20	3.925	22	3.864	23	3.597	23	3.408	24	2.168	29
Guizhou	3.574	26	3.749	24	3.784	24	3.957	21	4.125	22	4.292	20
Hainan	3.624	25	3.076	30	3.288	26	2.837	29	2.486	30	3.094	26
HeBei	6.077	10	6.097	10	6.465	9	5.972	10	6.183	10	5.523	16
Henan	6.483	9	6.615	8	6.919	8	6.633	8	6.670	8	7.020	8
Heilongjiang	4.015	22	3.825	23	3.902	22	3.369	25	2.927	28	1.074	31
Hubei	5.471	18	5.146	17	5.186	17	5.497	14	5.299	15	5.666	14
Hunan	5.944	11	5.410	14	5.705	13	5.702	13	5.605	13	5.646	15
InnerMongolia	5.513	17	5.226	16	5.230	16	4.814	18	4.631	20	4.562	19
Jilin	3.754	24	4.178	21	3.695	25	3.130	26	3.094	26	2.517	28
Jiangsu	12.304	2	12.144	2	12.613	2	13.185	2	12.735	2	10.434	2
Jiangxi	4.650	21	4.248	20	4.579	20	4.803	19	4.823	19	5.705	12
Liaoning	5.870	12	5.644	12	5.358	15	5.359	15	4.935	18	3.715	24
Ningxia	3.845	23	3.632	26	4.119	21	3.781	22	4.577	21	4.576	18
Qinghai	3.470	28	3.312	28	3.174	29	2.751	30	2.582	29	2.537	27
Shandong	10.240	3	10.057	3	10.552	3	10.773	3	10.417	3	8.718	4
Shanxi	5.660	14	4.901	19	4.713	19	4.689	20	5.127	16	6.074	10
Shaanxi	5.537	16	5.650	11	5.832	12	5.006	17	5.114	17	4.107	21
Shanghai	8.593	5	8.023	7	8.441	6	8.967	5	8.761	6	7.918	6
Sichuan	4.98	19	5.329	15	5.186	18	5.336	16	5.532	14	5.689	13
Sinkiang	3.537	27	3.716	25	3.197	28	3.003	27	2.967	27	3.888	22
Tianjin	7.986	7	8.204	6	8.420	7	7.132	7	6.763	7	4.944	17
Tibet	2.360	31	1.701	31	1.985	31	1.606	31	1.597	31	1.984	30
Yunnan	3.366	29	3.331	27	3.163	30	3.504	24	3.423	23	3.549	25
Zhejiang	9.496	4	9.280	4	9.951	4	9.691	4	9.395	4	8.206	5

**Fig 2:**
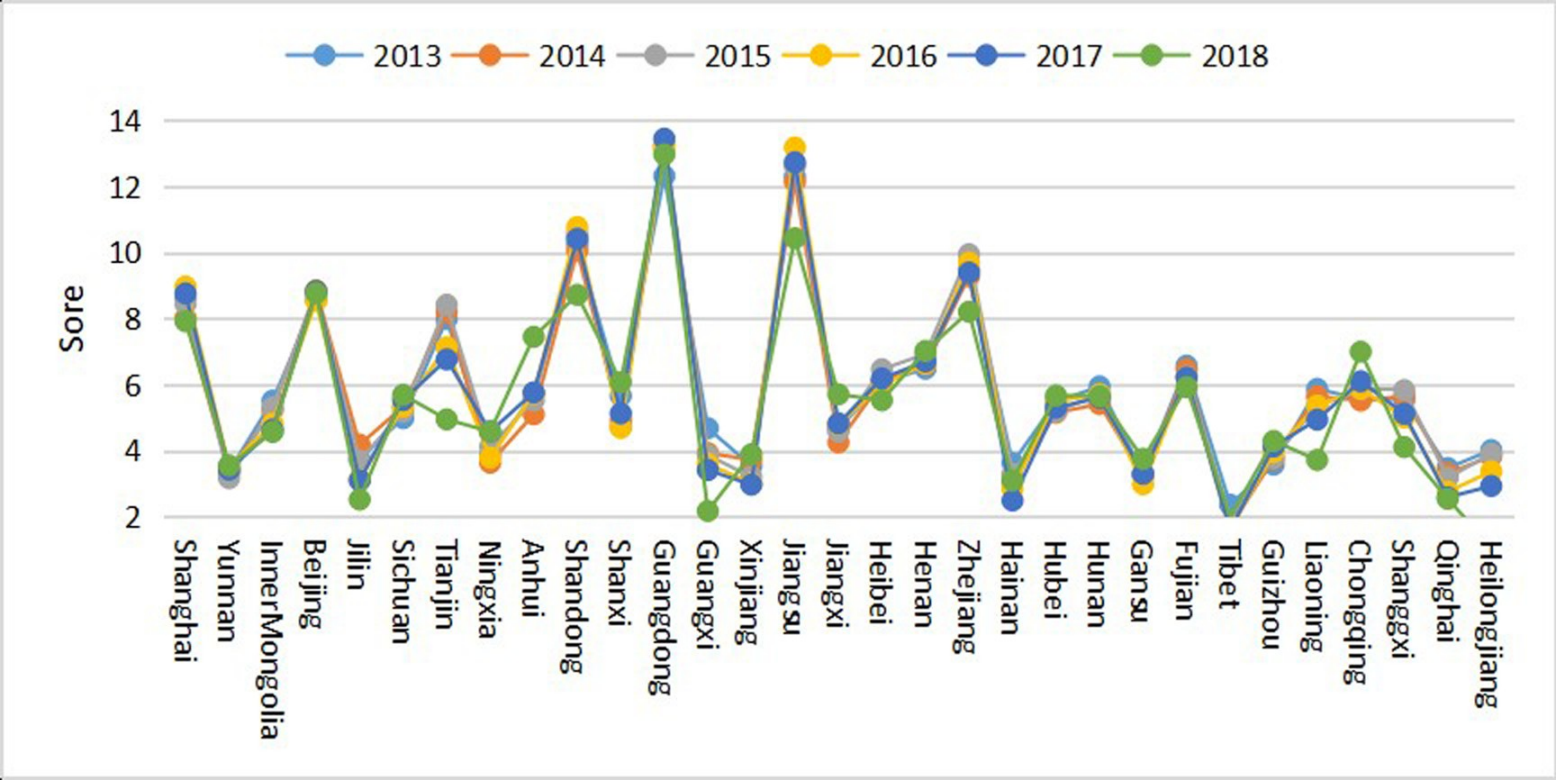
Change trend of GC scores of 31 provinces from 2013 to 2018.

Table 8 is drawn as a broken line statistical chart to facilitate observation, as shown in Fig 2.

Table 8 and Fig 2 show, from 2013 to 2018, the first-tier provinces of Beijing, Guangdong, Jiangsu, Shandong, Shanghai, and Zhejiang were at the top of the GC rankings. These regions ranked highest in the development of high-tech industries. Their Per capita sales revenue of high-tech new products and Per capita added value of manufacturing industry in high tech industry were high. They also ranked relatively high in the development of the Internet. Their E-commerce developed rapidly, and the green manufacturing capacity of MI was better. From 2013 to 2018, Jiangsu Province ranked second for GC, gradually replacing municipalities, and rapidly becoming the region with the strongest GC. Jiangsu is not a high gross product province. Still, its Utilization rate of industrial waste treatment is very high, its green manufacturing capacity is strong, and its degree of green development in regional manufacturing is high.

Jiangxi was a representative province of southeast China. Its GCMI rose from twenty-first place in 2013 to twelfth place. Its added value of the high-tech industry was high with significant development of the high-tech industry. Notably, its Internet penetration rate and E-commerce development index were close to those of the more developed areas. Environmental protection expenditure accounted for a large proportion of financial expenditure. This heavy investment in environmental protection gave Gansu Province a strong green manufacturing capacity. However, Per capita investment in science and technology was too small, and the number of R&D talents in regional manufacturing was less than in the more developed provinces and municipalities; therefore, the driving force behind green innovation remained insufficient.

Jiangxi and Gansu saw a relative faster growth rates among all the provinces. From 2013 to 2018, they rose 9 and 7 places, respectively, while Tianjin, Heilongjiang, and Guangxi dropped 10, 9, and 9 places, respectively. Hainan, Tibet, and Qinghai have been developing slowly. Although their regional manufacturing has the lowest levels of sulfur dioxide and sewage discharge, lacking development of the Internet, made their green innovation ability and green manufacturing ability the weakest. Also, their regional manufacturing markets were smaller, and their green market competitiveness was dim, resulting in the low GC of these three regions. Generally, although the GC of all regions in China has improved, the picture is mixed, as the overall level of green development was not high, requiring further enhancement of GC.

In the context of the new environmental and competitive pressures faced by MI, this study has introduced Internet application level and E-commerce development indexes, to construct an evaluation system for the scientific and comprehensive evaluation of the degree of development of the GC of provincial regional MI. Following Grey relational analysis of each index with entropy weight, the study finds that the Grey relational degree among the evaluation indexes is high, with a high relational state among the indexes. These results show that the new evaluation indicators introduced in this study are reasonable.

A ranking for GC in China’s provincial regional manufacturing from 2013 to 2018 was then calculated using the new evaluation index system. Given the entropy values for the period, the GC of China’s provincial MI improved continuously; however, the overall comprehensive score was low, with a wide regional development difference. The first-tier provinces (Beijing, Guangdong, Jiangsu, Shandong, Shanghai, and Zhejiang) tended to top the rankings for GC. The northwest and southwest provinces had fewer manufacturing enterprises and less sulfur dioxide and sewage discharge; however, their rankings were lower. Analysis of the data showed that these areas had less developed high-tech industry and lower per capita sales of high-tech products than the top areas with high competitiveness. And the numbers of B2B transaction volume were more limited than the top areas. These factors were responsible for the low GC of the provinces. However, Gansu, in the northwest of China, showed good development of high-tech industries, high E-commerce development index, and high levels of investment in environmental protection. The improvement in its rankings shows that green market competitiveness, green manufacturing ability, and green innovation ability have an important influence on the development of regional manufacturing and the cultivation of GC.

### 4.3. Spatial agglomeration effect

**Fig 3:**
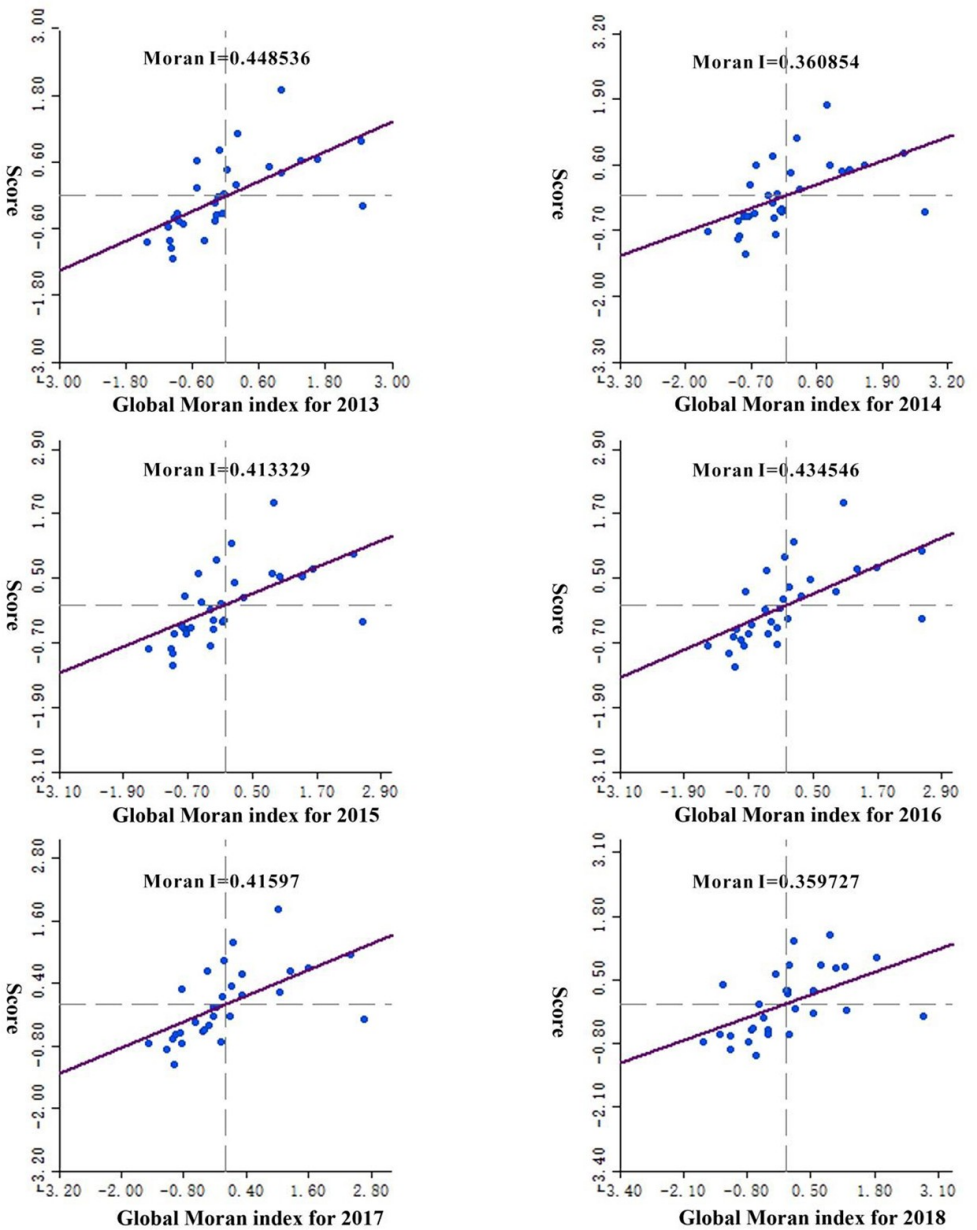
Global Moran index, 2013–2018.

Using the GeoDa spatial modeling software platform and Moran’s *I* index, the GC entropy weights of the 31 provinces from 2013 to 2018 were analyzed, and the global Moran index was obtained (Fig 3).

The global spatial Moran index reflects the situation of spatial agglomeration. The analysis results show that the global Moran index from 2013 to 2018 is all positive, and the Moran index is between 0.3597 and 0.4485, indicating that the GCRMI of each province in China was positively correlated in space and had agglomeration effect. From the perspective of time, the Moran index value from 2013 to 2018 first increased and then decreased, indicating the GCRMI agglomeration effect first strengthened and then weakened. The number of provinces and municipalities in the first and third quadrants has not changed much in the six years. The GCRMI of each province in each year has an obvious and stable agglomeration effect.

**Fig 4:**
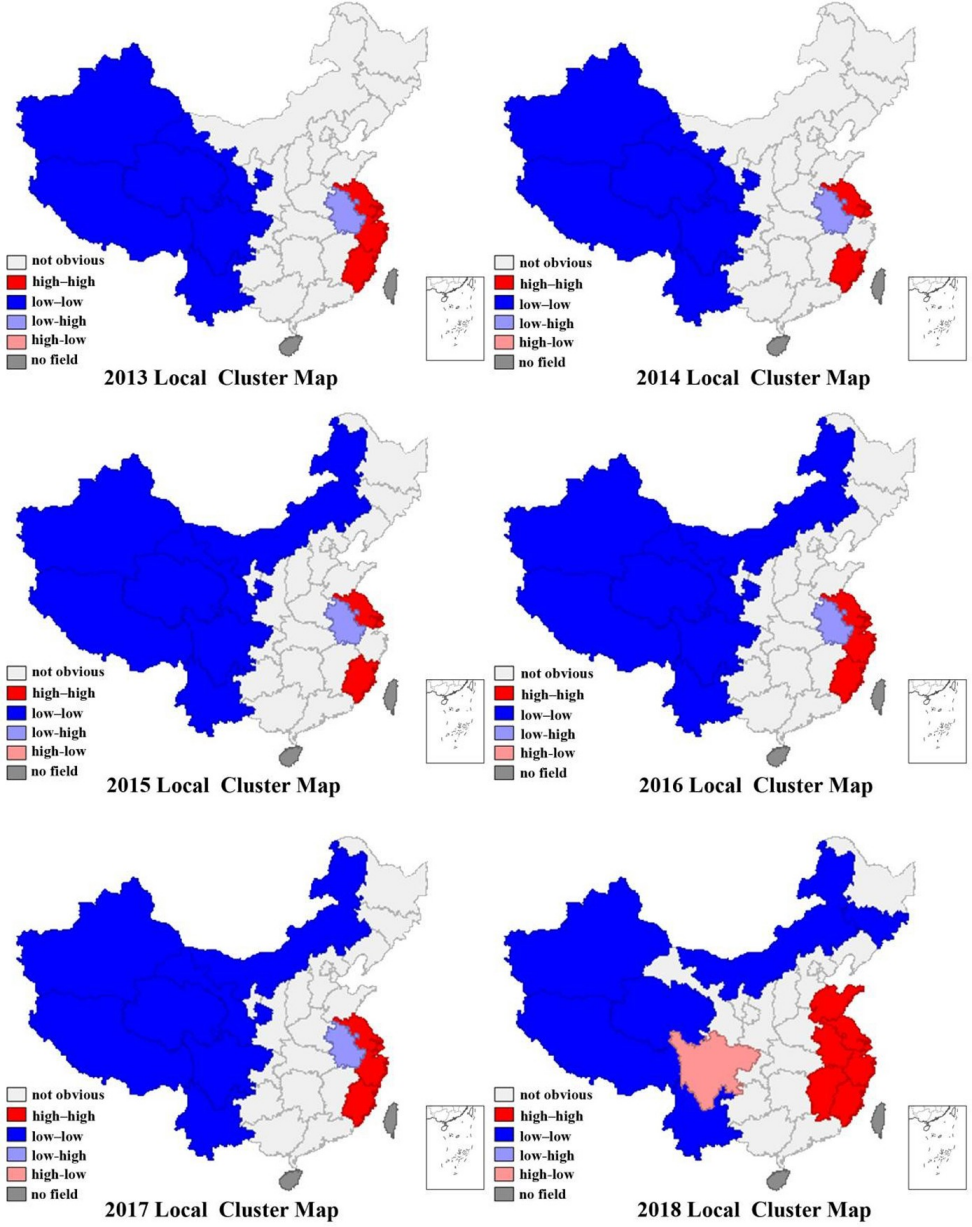
Local clusters, 2013–2018. Note: As it is difficult to obtain relevant data for Hong Kong, Macao, Taiwan, and Hainan Island. This study does not analyze these four regions.

The LISA cluster map chart for 2013 to 2018 was generated using GeoDa software from the graphical results in Fig 4. (The significance test value was set to 5%).

We use the Geoda software to test the local spatial autocorrelation from 2013 to 2018. From the results, the local spatial effect in 2013 was manifested as a clear "low-low" agglomeration in the western region, mainly distributed in Tibet, Xinjiang, and Gansu, Qinghai, Sichuan, Yunnan, and Ningxia; the eastern coastal areas show high-high agglomeration, mainly distributed in Fujian, Zhejiang, Shanghai, Jiangsu; Anhui region shows "low-high" agglomeration. Compared with 2013, 2014 lacked the “high-high” agglomeration in Zhejiang. In 2015, compared with 2014, the “low-low” agglomeration area in Inner Mongolia was increased. In 2016, the “high-high” agglomeration area in Zhejiang was added on the basis of 2015, and it remained unchanged until 2017. In 2018, there have been significant changes. The "low-low" clusters are mainly distributed in Xinjiang, Tibet, Qinghai, Yunnan, Inner Mongolia, and Jilin; the "high-high" clusters are distributed in Shandong, Jiangsu, Shanghai, Zhejiang, Fujian, Jiangxi, and Anhui; "High-high" agglomeration and "low-low" agglomeration have obvious differences. Sichuan is a "high-low" agglomeration area.

On the whole, the GC of China’s MI as a whole has a significant agglomeration effect. Specifically, the GCMI in the underdeveloped western regions has a low-low agglomeration. Tibet, Xinjiang, Qinghai, Yunnan, and Ningxia have been Located in the "low-low" agglomeration area, Shanghai, Fujian and Zhejiang have been in the "high-high" agglomeration. The development of China’s regional manufacturing green competitiveness is relatively stable in these regions.

## 5. Conclusions and implications

This study designed and constructed an index system for evaluating the GCRMI, incorporating three perspectives: green market competitiveness, green manufacturing ability, and green innovation ability. We selected 31 provinces in China from 2013 to 2018 as samples, and calculated the GCRMI and evaluated it using the Grey relational analysis method based on entropy weight. The major contribution of this study is the introduction of the Internet application indicators and E-commerce development index into the evaluation system for the GCRMI. The following conclusions can be drawn:

1. Grey relational analysis method based on entropy weight, which involves Internet application indicators and E-commerce development indexes for GC evaluation, is suitable for the new era.
2. The first-tier provinces (Beijing, Guangdong, Jiangsu, Shandong, Shanghai, and Zhejiang,) tended to top the rankings for GC. Gansu showed good development of high-tech industries, high E-commerce development index, and high levels of investment in environmental protection. The fastest growth rates were reported in Jiangxi and Gansu, while Tianjin, Heilongjiang, and Guangxi dropped in the ranking.
3. There was obviously spatial agglomeration effect GC of China’s integral manufacturing industry

### 5.1. Theoretical implications

Our study has several theoretical implications and contributions that are relevant to the theory of green manufacturing and GC. And the integration of the Internet and manufacturing industry is also studied. Thus far, It has rarely been treated within the framework of the evaluation index system or at the level of provincial MI. The indicators of the evaluation system developed here had high relational correlations. In particular, Internet penetration rate and E-commerce development indexes were at the top of the rankings, proving the importance of their effects on GC and offering useful insights into how the Internet and manufacturing might be integrated into the research on GC drivers and cultivation modes.

Our study also identified new sources of GC in Industrial Internet applications and diffusions, which constitute a new stream of supply chain management and production mode innovation. Indicators related to energy consumption and R&D capacity also ranked highly in terms of the degree of correlation. Our results confirm that environmental regulation and regional green innovation are two vital drivers of the GCMI. Our results are also likely to be useful for the development of GC in other industries.

The insights this study provides into the agglomeration of the GCRMI will promote the study of regional equilibrium in the GC of the manufacturing industry that is under pressure from national environmental regulations. It may also shed new light on development modes, paths, solutions, and policies for GC, not only for regional MI but also for other industries.

### 5.2. Practical implications

Our findings have important practical implications that will help policy-makers and decision-makers in manufacturing enterprises to make balanced and effective decisions promoting the GCRMI.

First, governments and manufacturing enterprises should enhance the manufacturing ability to be environmentally friendly, reduce pollution and emissions, and improve green manufacturing capacity. Therefore, it is necessary to avoid the persistent trend of excessive energy consumption in regional manufacturing production processes, and increase investment in technological renovation and technological innovation within MI. Taking these steps will improve the green production capacity of regional MI, thereby enhancing its GC.

Second, governments and enterprises should take advantage of the development of the Internet industry to improve the capacity of regional MI for green innovation and enhance its green market competitiveness. They also need to break the bottleneck of no core technology and no core products in Industrial Internet fields such as industrial software and industrial control security, to promote the manufacturing industry to be “high value-added,” “high-tech,” “intelligent” and “green,” to make the MI greener and more competitive. To enhance the GC of regional manufacturing, the concept of development will focus on “Internet + manufacturing” innovation and on improving green innovation capability combined with green demand.

Third, governments and enterprises should promote the balanced development of the GCRMI by sharing factories and new manufacturing ecosystems. The new green manufacturing ecology of technology-sharing and value-sharing can help cross-regional manufacturing to establish a more coordinated, efficient, and distinctive GC.

### 5.3. Limitations and further research

This study used provincial data from official statistics for the years 2013 to 2018, resulting in two limitations. First, GC was evaluated more indirectly than it would have been using data collected from companies. Second, the cultivation of GC takes a long time, and the aggregation effect in regions also needs a certain time to manifest. Therefore, the availability of only six years of data may have led to temporal inadequacy in observing and measuring these particular aspects of GC. From 2013 to 2018, the integration of the Internet and manufacturing had just begun. The Industrial Internet was also in its early stages in China. Their impact on green production and GC was not yet fully apparent, and this may have created uncertainty regarding Internet applications and other indicators. Therefore, future research should construct a new evaluation index system using indicators derived from the operation and performance of companies. Building on the results in this study, researchers can explore modes, paths, and more effective policies for achieving a national equilibrium in the GC of provincial manufacturing. Future research can also focus on strategies that companies can use directly to build GC in the context of the integration of the Internet and manufacturing at the level of industry or enterprise.
